# GHG Emissions from Dairy Small Ruminants in Castilla-La Mancha (Spain), Using the ManleCO_2_ Simulation Model

**DOI:** 10.3390/ani12060793

**Published:** 2022-03-21

**Authors:** Gregorio Salcedo, Oscar García, Lorena Jiménez, Roberto Gallego, Rafael González-Cano, Ramón Arias

**Affiliations:** 1 Centro Integrado de Formación Profesional (CIFP) “La Granja”, Barrio La Estación, 25-B, 39792 Medio Cudeyo, Spain; gregoriosal57@gmail.com; 2 Asociación Nacional de Criadores de Ganado Ovino Selecto de Raza Manchega (AGRAMA), Avda. Gregorio Arcos, 19, 02005 Albacete, Spain; oscargarcia@agrama.org (O.G.); rgallego@agrama.org (R.G.); 3 Instituto Regional de Investigación y Desarrollo Agroalimentario y Forestal de Castilla-La Mancha (IRIAF)—Centro Regional de Selección y Reproducción Animal (CERSYRA), Avenida del Vino, 10, 13300 Valdepeñas (Ciudad Real), Spain; ljimenez@quesomanchego.es (L.J.); rarias@jccm.es (R.A.)

**Keywords:** simulation model, sheep, goats, milk, carbon footprint

## Abstract

**Simple Summary:**

Greenhouse gas emissions from ruminants contribute to global warming. “ManleCO_2_” is an empirical model that simulates different management aspects in dairy sheep and goat farming, linking milk production to farming and environmental health. The carbon footprint of 1 L of fat- and protein-corrected milk varied from 2.01 to 5.62 kg CO_2_e. Simulation scenarios showed a higher reduction in GHG emissions associated with animal feeding strategies and a lower reduction associated with farming management strategies. ManleCO_2_ may provide useful information for planning and developing different strategies that might support the reduction of GHG emissions at the dairy sheep and goat farm level.

**Abstract:**

The first goal of this work was the description of a model addressed to quantify the carbon footprint in Spanish autochthonous dairy sheep farms (Manchega group), foreign dairy sheep farms (foreigners group: Lacaune and Assaf breeds), and Spanish autochthonous dairy goat farms (Florida group). The second objective was to analyze the GHG emission mitigation potential of 17 different livestock farming practices that were implemented by 36 different livestock farms, in terms of CO_2_e per hectare (ha), CO_2_e per livestock unit (LU), and CO_2_e per liter of fat- and protein-corrected milk (FPCM). The study showed the following results: 1.655 kg CO_2_e per ha, 6.397 kg CO_2_e per LU, and 3.78 kg CO_2_e per liter of FPCM in the Manchega group; 12.634 kg CO_2_e per ha, 7.810 CO_2_e kg per LU, and 2.77 kg CO_2_e per liter of FPCM in the Foreigners group and 1.198 kg CO_2_e per ha, 6.507 kg CO_2_e per LU, and 3.06 kg CO_2_e per liter of FPCM in Florida group. In summary, purchasing off-farm animal feed would increase emissions by up to 3.86%. Conversely, forage management, livestock inventory, electrical supply, and animal genetic improvement would reduce emissions by up to 6.29%, 4.3%, 3.52%, and 0.8%, respectively; finally, an average rise of 2 °C in room temperature would increase emissions by up to 0.62%.

## 1. Introduction

Small ruminants account for 56% of the domestic ruminants in the world [1]. They provide 15 million tons of meat and 25.5 million tons of milk [1]. Spain accounts for 23.6% of the EU sheep census and 22.2% of the EU goat census [2], contributing to 9.5% and 20% of EU milk production, respectively. Castilla-La Mancha, with 21.1% of the existing Spanish sheep farms and 15.5% of the Spanish sheep census, represents 32% of the national sheep sector. This region has 10.8% of the current Spanish goat farms and 14.5% of the Spanish goat census, representing 15.9% of the national goat sector [3].

The Manchega sheep is the Spanish autochthonous dairy sheep breed with the highest census presence, representing 78.7% of the sheep census in Castilla-La Mancha, followed by the Lacaune (16.0%) and Assaf (5.3%) breeds. Conversely, the Florida goat breed represents 3.7% of the regional goat census, according to data published by the Department of Agriculture, Water, and Rural Development in Castilla-La Mancha (Spain).

The Manchega sheep breed is well adapted to Castilla-La Mancha’s extreme climatic conditions; however, its milk production is lower than that of other foreign sheep breeds, such as the Lacaune and Assaf breeds, which are intensively reared in permanent housing [2]. However, in the last few years, the Manchega sheep breed has turned to be intensively reared, following more specialized farm management [4]. The replacement of grazing by supplementary off-farm feed and forage was the main step toward farming intensification [5]. These changes in the farming system (foreign breeds and intensive management practices) might give rise to negative environmental effects, such as an average temperature rise, water scarcity, water eutrophication, or soil loss, among others.

The most relevant GHG emissions from ruminants that are contributing to global warming are carbon dioxide (CO_2_) from fossil fuels and from changes in land use, methane (CH_4_), which is a physiological consequence of enteric and manure fermentation, and nitrous oxide (N_2_O) from manure management, fertilization, and the nitrification and denitrification process [6]. Although current emissions are high, there is great potential for reducing them. Gerber et al. [7] estimated that livestock farming was responsible for the production of 7.1 gigatonnes of CO_2_e per year, highlighting that 30% of these emissions might be reduced. However, the constant rise in livestock farming productivity might lead to a fall in emissions, mainly in terms of CH_4_ emissions, which chemical is the main greenhouse gas produced by ruminants [8].

Improving forage quality, reducing grazing time, and increasing the use of concentrated feed and genetic improvement [9,10,11] were the main options for increasing livestock farming intensification, among others. Although studies on livestock genetic improvement have not considered those aspects relating to the reduction of CH_4_ emissions or to efficiency in the use of nitrogen, there is no evidence of significant differences among different livestock breeds [11,12].

The experimental challenges associated with GHG emissions were discussed by Sanjo Jose [13]. Among others, an accurate estimation of the future effects of enteric fermentation, genetic improvement, and manure management on future climatic scenarios would require expensive and complex equipment and also years of work. Prevalent climatic conditions in any geographical location are a crucial factor affecting GHG emissions. Hence, huge research efforts would be necessary in order to identify profitable and less time-consuming strategies that could be applied broadly [13]. Considering that this type of expensive study should be repeated several times, modeling might be an efficient and appropriate alternative. In fact, several models have been developed to analyze livestock farming in different environmental scenarios, among them: MITERRA-Europe (based on the GAINS model: greenhouse gas and air pollution interactions and synergies); CAPRI (common agricultural policy regionalized impact) [14]; IMAGE (integrated model to assess the global environment) [15]; FarmGHG [16]; DairyWise [17]; SIMS-Dairy (sustainable and integrated management systems for a dairy production model) [18]; FARM-SIM (farm simulation model) [19]; IFSM (integrated farm system model) [20]; GLEAM-i (global livestock environmental assessment model—interactive) [21]; FarmAC [16] and DairyCant [22]. However, a scarce number of models have been developed to analyze the impact of domestic small ruminants on the environment; among them, LEITPA stands out as a model to analyze the environmental impact of sheep farming for meat [23,24,25].

To our knowledge, there is no single comprehensive modeling approach on the livestock farming scale that integrates the different elements that define sustainability, and, at the same time, that is capable of generating specific climatic scenarios linked to plant and animal production, economics, product quality, and process quality. The availability of a greater number of models would allow researchers to analyze environmental, nutritional, or economic trends from different approaches, which would guarantee a greater potential for the decision-making process regarding the feasible adaptative measures that are already taken or that will be taken in the future.

Usually, models requiring a large number of inputs are complex, resulting in accessibility problems that make them difficult to use, leading to losses and a reduction in their usefulness. This model was designed to be managed by public administration technicians, cooperative technicians, farm advisers, agronomists, vets, extensionists, researchers, agronomy students, etc. On the other hand, a strategic advisory plan would contribute to mitigating the negative effects of climate change through the implementation of the best available farming techniques.

This paper describes the ManleCO_2_ model as a simulation framework at the livestock farm level, assessing the potential for reducing the GHG emissions associated with 17 different management practices in dairy small ruminant farms in Castilla-La Mancha (Spain).

## 2. Material and Methods

### 2.1. Farms and Questionnaires

A total number of 36 small ruminant farms were surveyed during 2020: 25 of them correspond to autochthonous Manchega sheep, 6 farms to foreign breeds (Assaf and Lacaune), and 5 farms to the Florida goat, with all of the farms located in Castilla-La Mancha. They were analyzed using ManleCO_2_, taking the mean values of each herd (Manchega, foreign, and Florida) as a “baseline”, in order to generate different scenarios regarding the carbon footprint. The sample of the study represented 2.16% of sheep farms and 0.74% of goat farms registered in SITRAN (the Spanish animal traceability integral system) on 1 July 2020 [3]. In situ interviews included questions relating to: (i) location, (ii) land base and forage distribution, (iii) fertilization; (iv) breed composition and management, (v) animal feeding, (vi) the production and chemical composition of milk, (vii) equipment, and (viii) energy purchase.

All subjects gave their informed consent for inclusion before they participated in the study. The study was conducted in accordance with Regulation (EU) 2016/679 of the European Parliament and of the Council of 27 April 2016, regarding the protection of natural persons in terms of the processing of personal data and on the free movement of such data, repealing Directive 95/46/EC (general data protection regulation) and following the recommendations and instructions of the Spanish Protection Data Agency, according to the Spanish Organic Law 3/2018 on the protection of personal data.

### 2.2. Simulation Model Description

ManleCO_2_ is an empirical model based on research and statistical analysis. It offers a whole-farm balance perspective that simulates different management production models and the environmental health of dairy sheep and goats at the farm level.

Environmental health is calculated considering carbon footprint (including carbon sequestration), water footprint, and the emissions associated with land use, total and reactive nitrogen (N) footprint, energy footprint, acidification, eutrophication potential, and N and P (phosphorus) surpluses.

**Figure 1:**
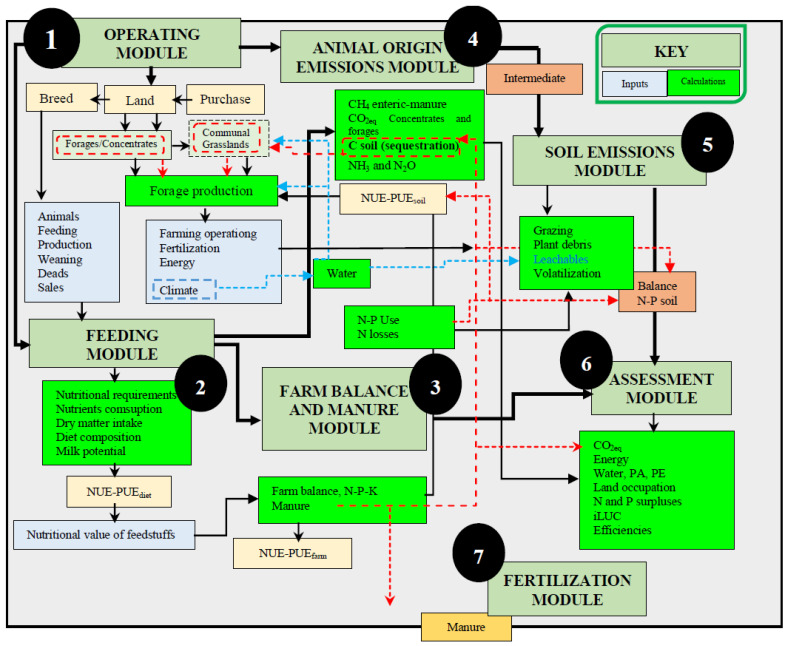
The ManleCO_2_ simulation model.

The model includes seven modules: (i) farm; (ii) animal feeding; (iii) farm and manure balance; (iv) emissions from animals; (v) emissions from the soil; (vi) assessment; and (vii) fertilization. Emissions limits and sustainability indexes are shown in Figure 1 and some of the algorithms that have been used are shown in Table 1.

The functional units (FUs) used by ManleCO_2_ are one hectare, one livestock unit (LSU), one present female (Pf), and one liter of fat (6.5%)- and protein (5.8%)-corrected milk (FPCM) [26]. Figure 1 represents the system’s limits, excluding equipment, buildings, and medicines.

The different pathways of the seven ManleCO_2_ modules, as well as interactions related to livestock management, climate, and production system at farm level, are shown in Figure 1:

- The farm’s forage potential and its intended use (hay, silage, or grazing).
- Input balance and outputs of nitrogen (N), phosphorous (P), and potassium (K), as well as potential losses in the soil–plant–animal system.
- Animal nutritional requirements; potential pasture consumption; N and P efficiencies; the excretion of N, P, K; and manure production.
- GHG assessment and potential carbon storage by the soil.
- Assessment of the farm’s environmental indicators, such as eutrophication and acidification potential, the whole N footprint, reactive N footprint, energy footprint, water footprint, and land use.

Information sources for modeling ManleCO_2,_ linked to management, productivity, animal feeding, forage production and climatology, among others, were provided by the Regional Center for Animal Selection and Reproduction in Valdepeñas (IRIAF-CERSYRA), the National Manchega Sheep-Breeders Association (AGRAMA), Castilla-La Mancha Department of Agriculture, Water and Rural Development, Integrated Vocational Training Centers, the Castilla-La Mancha Climate Change Office, DairyCant models [22], Cropwat 8.0 [27], and scientific references from the different journals that are mentioned throughout this paper.

### 2.3. Criteria and Steps for Modeling

Modeling was performed using the statistical software SPSS V21.0 for Windows (SPSS Inc., Chicago, IL, USA), according to the following criteria:

(i) Selected independent variables should be either easily measurable or information about them should be readily available. An animal model was designed using the number of sheep as an independent variable. Regarding the calculation of manure and urine volumes, as well as the daily excretion of N in terms of feces and urine, the considered independent variables were: supplementation (volume of forage or off-farm concentrates), volume of daily ingestion of the diet or forage, or their chemical composition (dry matter (DM); nitrogen (N); neutral detergent fiber (NDF); acid detergent fiber (ADF); organic matter digestibility (OMD); ethereal extract (EE) and starch), DM ingestion (g/kg of live weight^0.75^), feeding level, the percentage of forage and concentrates in the diet, and the in vivo or in vitro digestibility of DM, ODM, NDF and N. Regarding forage production, the considered independent variables were the sowing rate (kg of seeds/ha); sprouting time (days); the number of heat units (expressed in growing degree-days [GDD], according to [Equation (1)] and considering that base temperature equals to 4 °C) [28]; rainfall (mm per month); the number of days from seed to harvest; sprouting time (days); basal dressing (kg of N-P-K per ha); side dressing (kg of N per ha); seeds (kg per ha); sprout length (cm); total stubble and straw production (kg per ha); grain/straw ratio (%); and harvested straw (kg per ha).
(ii) The variables included in the models should be significant and highly correlated.
(iii) The model should fulfill all the assumptions of multiple regression analysis.
(iv) The model should have a high determination coefficient and a low standard error.
(v) The model should have low multicollinearity.

Heat units = [(T_MAX_ + T_MIN_) / 2] − T_BASE_ (1)

The different multiple regression models were analyzed following the methodology of “step by step”, accepting the result with the highest *R^2^* value, a minimum required signification level of 5% and no collinearity, using for this purpose the variance inflation factor ($VIF=\frac{1}{1-R_{i1}^{2}}$), which measures the variance dispersion of the x and y regressors, assuming 10 as the maximum cut-off value [29], and the Durbin–Watson statistic (a statistic that detects autocorrelation) to be set at lower than or close to 2. Besides this, two coefficients were studied (standardized and non-standardized) in order to evaluate the impact of every independent variable on the dependent variable, along with the relative importance of the independent variables.

Where experimental information was available, based on nutritional balances from sheep in metabolic chambers (feces excretion, urine volume, and N), based on the information available about the production of winter cereal crops used as forage (oats and triticale) or based on livestock farm management information, it was validated, according to the observed and simulated values, using five statistical indexes:

(i) Determination coefficient.
(ii) Concordance index “d”, as a standardized measure of the degree of error of the model prediction (considering that it can vary from 0 to 1, it acts as a dimensionless statistical index). A value equal to 1 indicates a perfect clustering between the observed and the simulated values; conversely, a value equal to 0 indicates that there is no clustering [30].
(iii) Root mean square error (RMSE), acting as a measure of the differences between the observations and the predictions [31].
(iv) The mean bias error (MBE) shows the systematic deviation [31]. When the MBE has a negative value, this indicates the model’s underestimation; conversely, a positive value indicates an overestimation.
(v) Model efficiency (EF), according to Nash and Sutcliffe [31], can vary from −1 to 1. When the EF value equals 1, this indicates a perfect coincidence between the simulated and the observed values; conversely, EF values of lower than 0 show that the average of the observed values would be a better predictor than the simulated values.

### 2.4. Modular Components of ManleCO_2_

#### 2.4.1. Operating Module (MEx_CO2_)

##### Animals

This comprises lactating animals (groups of high, medium, and low production), breeding animals, and studs. The stocking level is expressed in livestock units (LU) per hectare [32]. The quantification of non-adult animals and their intended uses (including adults) are shown in Table 1. In this study, 35 days is considered to be the average lactation period for offspring, with a daily intake of one L of milk per lamb. For farms using automatic lamb milk feeders, “marketed milk” refers to the milk produced by lactating females after the sixth day after the lamb is born.

The live weight of Manchega adult sheep, replacements, and lambs intended for slaughter (at 35 days of age, approximately) was fitted to the Gompertz model, considering a sample of 272 animals aged between 2 and 480 days, from four farms, extrapolating that curve to the one belonging to foreign breeds. Conversely, in the case of foreign adult goats, replacement females, and kids intended for slaughter, the Gompertz model was used, according to the methods in [33] for Alpine and Saanen breeds. We fitted the live weight established in the Florida breed standard for Spanish adult goats (55 kg).

##### Land, Purpose, and Production (Only Considering the Area Used for Feeding Animals)

The farming operations, fertilization, and production per hectare of grain cereals, alfalfa, and maize for silage were provided by the farmers. The DM production for fodder from winter cereals, oats, and triticale (Table 1) was based on the figures for grain cereals reported in Castilla-La Mancha by the Regional Department of Agriculture, Water, and Rural Development from 2013 to 2017. For this purpose, grain production was considered as being the sum of the stubble and the harvested straw, depending on the type of cereal [34], considered as forage until the beginning of ripening of the grains. The agronomic variables of every experiment (dose of N fertilizer per hectare (basal and top dressing), number of days for grains ripening, plant height, grain production, and climate information (provided by Castilla-La Mancha Climate Change Office)) were the bases for estimating their forage equivalences, considered as the potentially processable quantity of a cereal grain into fodder up to “leaf state flag”. Rainfall was used to estimate the value for N leaching in the soil emissions module (MEsu_CO2_), as well as the water footprint of the feed produced on-site at the farms in the assessment module (MEva_CO2_), using CROPWAT^®^ (FAO, Rome, Italy, 2009) [35,36].

The main sources of carbon incorporated into the soil were manure and plant debris, in terms of both stubble and roots. In this study, we considered the height for grain cereal stubble to be 16 cm and a production of 1758 kg of DM per hectare for straw [34] and for fodder cereals [34]. The maize biomass estimation was 19% of the whole harvest [36]. The alfalfa biomass estimation was lower than 34% of the whole harvest [37]. The sequestered carbon by the soil was estimated according to the method used in [38], assuming that plants biomass content was 45% [39], and in terms of manure content, a C/N ratio of 13.4% was assumed [40].

##### Energy

By default, ManleCO_2_ assumes a consumption of 37 L of diesel per year and LSU [41]; conversely, electricity supply (KWh) is a function of the number of lactating females and milking time (in minutes) [42].

#### 2.4.2. Feeding Module (MAlm_CO2_)

##### Feedstuffs

The model includes the chemical and bromatological composition of forages produced and sold in Castilla-La Mancha, using data from the DairyCant model [22] and FEDNA (Fundación Española para el Desarrollo de la Nutrición Animal) data for concentrates [43]. Energy values for silage, forage, feed, and mixed rations were taken from [44,45] and the k_L_ efficiency from [46].

##### Nutritional Requirements

ManleCO_2_ estimates the amount of net energy required for milk production (NE_MILK_) in terms of Mcal; the amount of metabolizable protein, in terms of grams, according to [47]; calcium and phosphorus [48] and neutral detergent fiber [49]. The theoretical needs were adjusted according to the following sheep production levels: (i) early growth (0–4 months old); (ii) growing–finishing (4–12 months old), (iii) breeding lactating females (0–60 days postpartum = high milk production batch; 60–120 days postpartum = medium milk production batch; 120–180 days postpartum = low milk production batch) and (iv) non-lactating females, including pregnant ewes. The needs were estimated in terms of live weight (kg), milk production (liters per day), fat (%), protein (%), number of offspring, lactation stage (days), age at first lambing, weight change (kg per day), grazing conditions (kilometers traveled per sheep and per day) and grazing time. The theoretical dry matter intake (DMI) was estimated on the basis of studies made in sheep [50] and goats [51], as well as studies about grazing in both species [52]. The potential milk production was calculated by subtracting the maintenance net energy (Mcal) [53] (grazing activity included) from the total milk energy concentration in the Manchega breed [54], Lacaune and Assaf breeds [55], and goats [56], divided by the net energy intake (NEI). Similarly, the potential from the ingested protein was evaluated, assuming assimilation rates of free amino acids in milk protein synthesis of 68% for goats [57] and 58% for sheep [58].

##### Nitrogen Use Efficiency (NUE) and Phosphorous Use Efficiency (PUE) in Animal Diets

Both are shown as the percentage of N and P excreted through milk and meat together, in relation to ingested N or P.

#### 2.4.3. Farm Balance and Manure Module (MEstNu_CO2_)

This module analyzes manure production and nutrient balance at the farm level.

##### N and P Balance at Farm Level

N and P inputs are calculated by summing up the purchased feed, fertilizers, animals, atmospheric N, bedding straw, and N fixed by legumes, and by subtracting the N and P outputs (from milk, wool, meat, and manure). Concentrations of 0.28 kg of N and 0.0065 kg of P per kg of sold or purchased live weight were assumed [51], respectively. The assumed value for the atmospheric N was 10 kg per hectare and year [59] and the value for N, fixed by legumes in the soil, was taken from [60]. Considering both marketed milk and milk intake by lambs/kids as a whole, N outputs from milk were calculated by dividing the crude protein value by 6.38, and the P outputs from milk were estimated at 1.3 g of P per liter [61]. In the case of wool, the N output was given a concentration value of 12.8% of its weight [62] and P output from wool was considered as 0.1 g per kg [63]. N and P concentrations in manure are detailed in the following section. N and P surpluses were estimated in kilograms per hectare, and they were calculated as the difference between inputs and outputs, while the efficiency was estimated in terms of a percentage.

##### Manure Production

Equations for calculating the manure production in terms of volume (feces and urine) and its N content are taken from 32 nutritional balances, carried out in a metabolic unit with sheep fed at maintenance level; P and K concentrations were assumed to be those given by [63].

The final manure production per hectare was calculated by subtracting the feces and urine excreted during grazing (estimated from grazing time and animals grazing throughout the year) to the sum of feces, urine, and the bedding straw (produced on-farm or purchased). The assumed concentration values for N, P, and K linked to grazing were 0.55%, 0.07%, and 1.1%, respectively. In addition, 225 kg of bedding straw per female and per year was considered in the study [64].

N losses after manure application are only considered when the manure is used at farm level as a natural fertilizer. By contrast, when manure is sold, only NH_3_ losses at barn level are to be considered. Similarly, for the calculation of carbon sequestration, it is assumed that only the unsold manure is poured over the entire farmland, including communal lands.

#### 2.4.4. Animal Origin Emissions Module (MEoa_CO2_)

Enteric CH_4_ and manure emissions were estimated according to [65]. The total net energy requirement and the digestible energy of the feed were used to calculate the gross energy requirement and the feed intake. Methane emissions from enteric fermentation were calculated by applying a methane conversion factor. The IPCC (2006) established that the CH_4_ conversion rate (Ym) was equal to 6.5, although it could vary depending on the digestibility of the diets and the productive stage of the animals. Ym was calculated according to [Equation (2)]. Enteric CH_4_ (in terms of grams per sheep and day) was calculated according to [Equation (3)], where 18.55 is the value for contained gross energy in feedstuffs (MJ per kg of what is considered as the potentially processable quantity of a cereal grain into fodder, up to DM) and 55.65 is the value of energy provided by CH_4_ (MJ per kg).

For the calculation of CH_4_ emissions from manure [65], we assumed that manure has a DM of 35%. The value for NH_3_ content in barns and manure heaps was calculated from the excreted N (feces + urine), assuming an emission factor of 0.1 kg of NH_3_ per kg of excreted N [66] and, in the case of grazing, we assumed a value of an emission factor of 0.12 kg of NH_3_ per kg of excreted N per grazing days and per hectare [67].

The N_2_O emission factor for barns and manure heaps was estimated at 0.0015 kg of N_2_O per kg of excreted NH_3_ [68] and as 0.025 kg of N_2_O per kg of excreted N, minus excreted NH_3_ while grazing [66].

Ym = 9.75 − 0.05 × OMD (2)

Enteric CH_4_ = DM × (Ym/100) × (18.55/55.65) (3)

#### 2.4.5. Soil Emissions Module (MEsu_CO2_)

N_2_O emissions from the soil were classified as direct and indirect emissions. N_2_O direct emissions are associated with the distribution of manure, fertilizers, and plant residues, with emission factors of 0.003, 0.01, and 0.01 g N-N_2_O kg^−1^, respectively [69]. Applied manure is the difference between the manure produced at farm level (MEs_CO2_) minus the manure that is sold or excreted while grazing, multiplied by the stocking level, without any allocation to a given crop. Indirect emissions include NH_3_ volatilization (5 g N-N_2_O per kg of volatile N) [70] and leaching (25 g N-N_2_O kg^−1^ of leached N) after applying organic and inorganic N to the soil [70]. The leached NO_3_^−^ was estimated as a function of the total N applied per hectare and the volume of drained water, estimated from the evapotranspiration (ET_c_) value minus rainfall [71].

##### Animal Emissions, Soil Emissions, and Intermediate Calculations

Other indirect emissions to be considered are the purchased fertilizers, considering an emission factor of 5 g N_2_O per kg of N fertilizer [72], and the purchased forages and concentrates with emission factors of 20 g and 10 g of N per Kg of purchased N, respectively [70]. The NH_3_ emissions from mineral fertilizers and from manure were given a value of 0.01 kg of N_2_O per kg of NH_3_. The purchased goods and services are linked to CO_2_ emissions that are considered as intermediate ones (Figure 1), including diesel and electrical supply (3.31 kg of CO_2_ per liter and 0.65 kg of CO_2_ per KWh, respectively [73]. The electrical supply was calculated per ewe and per year [42]. For fertilizers, we assumed values of 6.2 kg of CO_2_ per kg of N, 0.93 kg of CO_2_ per kg of P_2_O_5,_ and 0.51 kg of CO_2_ per kg of K_2_O, respectively [65]; in the case of forage, we assumed values of 0.2 kg of CO_2_ per kg, and for feed, 0.3 kg of CO_2_ per kg [74]; for plastics, we assumed 2 kg of CO_2_ per kg [74]); purchased animals were assumed at 11 kg of CO_2_ per kg of live weight kg [74]; and for pesticides, we assumed an average value of 22.2 kg of CO_2_ per ha [74].

ManleCO_2_ considers the balance of N and P in the soil as intermediate calculations; they are calculated from the differences between inputs and outputs, in terms of kg per ha. N inputs are considered to result from the addition of the following sources: organic N + inorganic N + atmospheric N + symbiotic fixation of N + recycled N + N from a mechanical origin; P inputs are calculated as the sum of organic P + inorganic P + P from a mechanical origin. N outputs are the result of the addition of NH_3_ + NO + N_2_ + N_2_O + NO_3_ crop extractions, and finally, the only P output considered is the P extracted from soil. N and P utilization efficiency rates in the soil, in terms of percentage, are calculated as follows: [100 × (extractions − plant residues)] ÷ (inputs − balance).

#### 2.4.6. Assessment Module (MVa_CO2_)

##### Carbon Footprint in Milk and Meat

The functional units used in the study were 1 hectare, 1 LU^−1,^ and 1 L of corrected milk by fat and protein [26], expressed in CO_2_e, considering the next equalities: 1 CO_2_ = 1 CO_2_e_,_ 1 CH_4_ = 28 CO_2_e, and 1 N_2_O = 298 CO_2_e, in accordance with [65]. The partial carbon footprint (PCF) of every functional unit was considered as: Σ (CH_4_ + CO_2_ + N_2_O), and the total carbon footprint (TCF) as: Σ (PCF + SY + iLUC − CS). The total carbon footprint (CF) is equal to the partial carbon footprint (PCF) plus SY (emissions attributed to soybean) plus iLUC (indirect land-use change) minus carbon sequestration (CS). In addition to milk, sheep and goat dairy farms sell meat (lambs and kids, animals, and breeding animals), allocating a percentage of total emissions to both milk and meat [75]. The allocation of emissions to milk or meat production was estimated according to [Equation (4)] by [75], where *AF* stands for the allocation factor, *R* is equal to M meat / M milk, where *M meat* is equal to the sum of live weights of all animals sold per hectare and *M milk* is equal to the sum of the total weight of milk sold per hectare.

AF = 1 − 5.7717 × R(4)

Indirect Land Use Change (iLUC) and Soybean Emissions (SY).

Emissions from cultivated areas were allocated a value of 143 g of CO_2_ per m^2^ and per year [76] and 2.98 kg of CO_2_ per kilo of imported soybean [77].

##### Total Water Footprint (WF_t_)

The total water footprint is calculated as the sum of green, blue, and greywater [78,79], estimated from the water used in food production (water_fp_), water contained in feedstuffs (water_pf_), drinking water (water_d_), and cleaning water (water_c_). The water for the domestic production of forage was calculated according to CROPWAT^®^ 8.0 model by FAO (Rome, Italy) [35]. The green, blue, and greywater of imported products was estimated according to [80]; drinking water [81] was estimated by allocating 2.2 L of cleaning water per liter of milk. The sum of blue and grey waters is defined in this study as a partial water footprint (WFp).

##### Total Energy Footprint (EFt)

The total energy footprint is calculated as the sum of the direct (EF_di_) and indirect (EF_in_) footprint and is expressed in megajoules. EF_di_ is mainly diesel and electrical supply, and EF_in_ is associated with imported resources (fertilizers, feed, seeds, plastics, medicines, hired services, and herbicides) [82].

##### Acidification Potential (Ap) and Eutrophication Potential (Ep)

Acidification potential (*Ap*) is expressed in terms of SO_2_ equivalents (SO_2eq)_ [83], and considering that 1 SO_2_ = 1 SO_2e_; 1 NO_x_ = 0.7 SO_2e_ and 1 NH_3_ = 1.89 SO_2e_. Conversely, the eutrophication potential, *Ep*, is expressed as NO_3_ equivalents, considering that 1 NO_3_ = 1 NO_3e_; 1 NO_x_ = 1.35 NO_3e_; 1 NH_3_ = 3.64 NO_3e_ and 1 PO_4_^−^ = 10.45 NO_3e_ [84].

##### Total Nitrogen Footprint (NFt) and Reactive Nitrogen Footprint (NFr)

The total nitrogen footprint (NFt) represents the sum of the total imported N sources (purchased feedstuffs, purchased animals, fertilizers, biological nitrogen fixation, and atmospheric deposition). The reactive nitrogen footprint (NFr) is calculated as the addition of NH_3_, N_2_, N_2_O, NO, and NO_3_^−^ [85].

##### Land Use (Land Use_Off_, Land Use_On_, and Land Use_Total_)

Land use is expressed in terms of m^2^ per liter of fat (6.5%) and protein (5.8%) corrected milk (FPCM) [26] and was calculated according to off-farm feed and on-farm feed. Off-farm feed production, in terms of tons per hectare, were estimated as follows: 2.9 for barley, 2.5 for rapeseed, 10.6 for corn, 2.9 for soybean, 4.7 for beetroot pulp, 4.4 for cottonseed, 1.3 for sugarcane molasses, 3.0 for palm oil, 11.3 for alfalfa and 2.5 for cereal straw.

#### 2.4.7. Fertilization Module (MFt)

ManleCO_2_ tackles fertilization by using a separate module for every farm. Crop nutrient requirements are estimated on the basis of soil type, chemical composition, crop outputs, direct inputs (grazing excreta), and indirect inputs (manure and chemical fertilizers). The interpretation of soil analysis is based on the recommendations given by [86] and nutrient supply is defined on the basis of: (i) outputs (N, P, K, Mg, and Ca) and (ii) the maintenance of fertility parameters.

### 2.5. Simulated Scenarios

The “*baseline*” group (Table 1) was simulated with the average values from every surveyed breed: Manchega, foreigners (Lacaune and Assaf), and Florida (goats).

The simulated scenarios are focused on climate change and on the practices that the farmer has the greatest likelihood of choosing, in terms of emissions: (i) baseline; (ii) genetic improvement; (iii) animal inventory; (iv) purchased feed; (v) management forage; (vi) electrical supply, as is shown in Table 2.

The “genetic improvement” group (Manchega breed only) refers to the number of adult animals that can be reduced by increasing the genetic value (GV) of the flock without changing the final milk production. The genetic value of the National Manchega Breed Flock (GV_NMB)_ [88] was used as an independent variable to estimate milk production by lactation, obtaining [Equation (5)]; standard error (se) = 24.2; r^2^ = 0.89. The GV_final_ was calculated as the difference between the GV_initial_ (provided by AGRAMA) and the increase of each situation (5%, 10%, or 15%) to the GV_initial_.

Liters of milk per lactation = 198.3 + (5.3 × GV_NMB_) (5)

The obtained value, multiplied by the equation slope, represents the potential volume of milk produced per ewe and per lactation. This value was divided by the addition of marketed milk and milk intake by lambs, in order to determine the potential number of animals to be reduced.

The equation assumes that the environment is the same in all farms and that only genetic variation can have an effect on the higher or lower number of animals per farm.

The group “animal inventory” simulates those aspects related to the variation in the number of animals per group (lactating, non-lactating, and replacement females) and is related to management (dead animals), used as a strategy to minimize greenhouse gas emissions.

The “feed” group simulates the mitigation potential, which is related to changes at the animals’ diet level: ingredients, grazing, etc.

The group “forage conservation” simulates the emissions related to two different approaches: (a) 25% of the area sown with oats and vetch is preserved as hay; (b) the remaining 75% is preserved in silage bags or in small silos. The amount of plastic used per small silo was 1.3 kg [36] and, in the case of the silage bags, the amount of plastic was 0.68 kg per ton of silage, assuming in both cases that the emission factor for plastic is 2 kg of CO_2_ per kg [67]. The CO_2_ from silage fermentation processes was assumed to be 0.24 kg of CO_2_ per kg of lost dry matter [89]. The loss of dry matter was estimated as 12.3% for cereals silage and 3% for maize silage [90].

The “electrical supply” group simulates the mitigation potential by reducing milking time by 10%. The electrical supply was considered according to [42], depending on the number of lactating females, milking time in minutes, and KWh per female and year, assuming an emission factor of 0.65 kg of CO_2_ per kw/h [73].

The “climate change” group simulates variations in milk production linked to a raise of 2 °C in the average room temperature, according to the average increase of 0.12 °C per year during the period of 2000–2017 in the Castilla-La Mancha region. The National Manchega Breed Flock milk production figures and the content of fat and protein were linked to the average environmental temperature [91], and to the temperature–humidity index (THI) [92], giving rise to six different equations (cc_milk_-Tª; cc_milk_-THI; g_fat_-Tª; g_fat_-THI; g_protein_-Tª and g_protein_-THI) from three breeding seasons (April–May; July–August, and October–November).

Subsequently, considering the climatic data from the municipalities where the farms are located (Castilla-La Mancha Climate Change Office), an average temperature increase of up to 2 °C was simulated in order to estimate the loss in milk production and in fat and protein contents, linked to the increase in ambient temperatures, for every month within the breeding season.

## 3. Results

### 3.1. Farms

The technical and productive features of the three racial groups (Manchega (M), foreigners (F), and Florida (FL)) are summarized in Table 3. The agricultural land use was higher in group M. The agricultural land is used for the production of forage (winter cereals, a blend of cereals and annual legumes or a blend of maize for silage and legumes, mainly alfalfa) and grain cereals. In general, fallow land, grain cereals, and forages represent 40.8%, 26.4%, and 24.2% of the agricultural surface and, to a lesser extent, maize (3.3%) and legumes (9.7%), such as peas, vetch, or alfalfa. The average crop productions were 2.7 tonnes per hectare and per year for grain cereals (barley), 4.8 for fodder cereals (triticale and oats), 15.8 for maize, 15.7 for alfalfa, and 4.4 tonnes per hectare and year for annual legumes (peas and vetch). Annual chemical fertilization was not different among flocks, with average inputs of 23-6-5 kg per hectare and year (of N-P-K, respectively) over the whole area and 55-13-11 kg per hectare and year, respectively, in the case of the area under cultivation.

The number of adults was higher in groups M and F, while the stocking density was lower in groups M and FL (Table 3). At the farm level, lactating females represent 62.3% of the adults, which is higher in group F (83.7%) and lower in M (58.3%) and FL (56.1%), and they were distributed according to their milk production level, into high (28.6%), medium (18.1%), and low (15.5%) production lots. Animals for replacement (4–12 months) represented 26.9% of the censuses, and their number was higher in F (30.6%) and lower in FL (21.6%). The breeding female replacement ratio was different among breeds, varying between 0.21 in FL and 0.31 in F.

The purchase of concentrates per present female and year was higher in group F (Table 3), without any differences regarding forage. The land used for the production of the purchased feed (forage and concentrates) was different among racial groups, being higher in FL with 8.6 m^2^ per liter of milk (Table 3); 2.7 m^2^ corresponding to concentrates, and 6.1 m^2^ to forage, respectively. The higher amount of agricultural land used in M promoted higher levels of own-forage intake. The grazing time was longer in M, mostly in non-pregnant animals.

Milk production, corrected by fat and protein (FPCM) [26] per farm, per hectare, and present female, was higher in group F as well as in terms of live weight sold per hectare (Table 3). Milk production, given by LU, was 38.9% higher in group F compared to M. No differences in cheese extract production (kg per LU) were found between F and M. The live weights sold (kg per LU) were similar in M and F and lower in FL. Likewise, the production of FPCM per kg of ingested dry matter (DM) was similar in F and FL and lower in M (0.30 kg of FPCM per kg of DM; these increased up to 0.81 kg when only lactating females were considered). In addition, the nitrogen use efficiency at farm level (NUE_farm_), the milk yield from lactating dairy females (NUE_milk-lactating females_), and the sum of milk and meat from all animals together (NUE _milk+meat all animals_) were similar among breeds, with average values of 23.8% (M), 19.6% (F), and 32.9% (FL).

### 3.2. Development of an Animal Model, Excreta Production Model, and Forage Production Model

The animal model includes the number of replaced females, lambs and kids born, culled lambs and kids, losses, dead adult ewes, discards, deaths of lambs and kids, abortions, etc. The excreta production model includes feces and urine volumes, as well as the daily excretion in terms of N. The forage production model corresponding to triticale and oat is shown in Table 4, and in Table 5, the main characteristics of the independent variables of the model are presented.

The first model was developed according to the technical and economic management data provided by the National Manchega Sheep Breeders’ Association (AGRAMA), from the period of 2014–2015. The second model was the result of applying to forage production the results of different experiments carried out by the Castilla-La Mancha Department of Agriculture, Water, and Rural Development. The third model was developed, considering the results from different experiments carried out in the metabolic unit in sheep by the Integrated Center for Vocational Training in Heras (Cantabria, Spain).

Non-standardized coefficients related to the number of replaced females, born lambs and kids, culled lambs and kids, losses, dead adult ewes, discards, lambs’ and kids’ deaths, abortions, etc, showed increases of 0.42, 1.91, 0.98, 0.4 and 0.55, respectively, by increasing one present female per farm. Standardized coefficients related to the independent variable “present female” (Table 4) were high, as well as the determination coefficient (Table 5). The non-standardized coefficients for feces excretion indicated that an increase of one percentage unit in OMD would lead to a decrease in the feces per sheep and per day equal to 0.69 g in terms of DM, and to an increase of 0.08 g per gram of ingested DM, 0.57 g per gram of ingested gross protein, and 0.26 g per gram of ingested NDF.

Among the standardized coefficients, OMD was the most relevant; conversely, the less relevant was DM consumption (Table 4). N consumption, as an independent variable, explained 56% of the volume of urine that is produced, 78% of the N excreted in the feces, and 64% of the N excreted in the urine (Table 5). According to this finding, for every gram of N that is ingested, 71.3 mL of urine is produced, 0.24 g of N is excreted in the feces, and 0.64 g of N is excreted in the urine.

The non-standardized coefficients for triticale forage, as shown in Table 5, indicate that the yield per hectare increased by 133.2 kg of DM per every cm in plant height; also, that it could increase up to 33.9 kg of DM per every growing day in the plant up to the emergence of the ear, and up to 62.9 kg of DM per every kg of N applied as basal dressing.

The standardized coefficient of the independent variable “triticale plant height” (Table 5) appeared to be the best predictor by which to estimate the amount of kg of N to be applied as basal dressing and, also, to estimate the number of days up to the emergence of the ear in the plant. However, in terms of oat forage, plant height, as an independent variable, explained 87% of the harvested biomass. According to this finding, every cm of increase in plant height could lead to an increase of 112.6 kg of DM per hectare (Table 5).

All the variables showed variance inflation factor (VIF) values under 10 and lower values of the Durbin–Watson statistic (DW), in any case, that were close to 2 (Table 5), as well as acceptable values of the coefficient of determination except in “lambs and kids deaths” (r^2^ = 0.59), “abortions” (r^2^ = 0.56) and the volume of urine (ml). Generally, simulations of the models showed low RMSE (root mean square error) scores, low MBE (mean bias error) scores, acceptable ME (model efficiency) scores always at greater than zero, and a high index of agreement (d). In any case, MBE scores that were higher than zero indicated an overestimation of the variables analyzed, and conversely, the negative scores highlighted their underestimation.

### 3.3. Potential Mitigation in Different Scenarios

**Figure 2:**
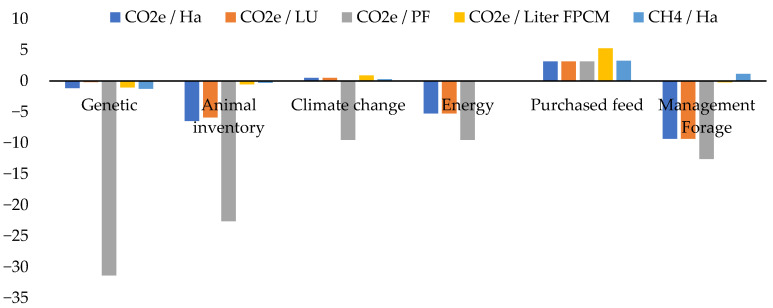
Simulated strategies: changes among groups in terms of the average percentage of emissions.

The effects of management changes on the total emissions per hectare, per livestock unit (LU), and per liter of fat- and protein-corrected milk (FPCM) from each simulation are shown in Table 6 and Figure 2 for all breeds in the study. By increasing 10% of the genetic value of the Manchega breed, the number of adult animals could be reduced; in turn, milk production could be maintained, and emissions could be reduced by 1.45% per hectare, 0.65% per LU, and 1.37% per liter of FPMC. Culling 10% of the unproductive sheep (discards) and reducing replacement by 5% could lead to a reduction in emissions of up to 1.47% per hectare and 1.85% per liter of FPCM, increasing 1.01% per LU.

Animal feeding showed a high mitigation potential in all breeds. For instance, and related to feedstuff purchase, the replacement of soybean by peas in the concentrates could reduce emissions by up to 16.7% per hectare, LU, and liter of FPCM. Conversely, the replacement of conventional concentrates by other ingredients based on by-products could increase emissions by up to 19.1%. Conversely, the replacement of breastmilk by formula could increase emissions by up to 7.0% per hectare and up to 13.7% per liter of FPCM.

Forage management plays a fundamental role in the mitigation of emissions. When replacing oat hay with another feedstuff with a higher relative forage value (RFV), emissions could be reduced by up to 10.2% per hectare and LU and by 4.6% per liter of FPMC. Nevertheless, these percentages might be higher when considering the potential increase in milk production. In the case of the Manchega breed, grazing spring triticale for 100 days led to a decrease of 14.3% and 0.4% CO_2_e emissions per hectare and per liter of FPMC. However, when grazing triticale was combined with a 15% reduction of the concentrates in the animal diet, emissions were reduced by only 9.8% compared to the previous figures (Table 6). The conservation of forage in silage bags can lead to a reduction of 3.16% of emissions in all breeds, and only to a reduction of 0.62% when using silage round bales (Table 6).

A reduction of 10% in milking time could reduce emissions by up to 15.5% per hectare and 0.12% per liter of FPCM in goats and 0.11% per hectare and 0.12% per liter of FPCM in sheep.

On the contrary, a rise of 2 °C over the average room temperature in all the breeds could lead to an increase in emissions of 0.48% per hectare, 0.48% per LU, and 0.89% per liter of FPCM, as a result of lower milk production.

## 4. Discussion

### 4.1. Excreta Production Model

N intake is a very useful independent variable for the estimation of urine and fecal N excretion in sheep [93,94]. Patra (2010) analyzed these two parameters and he obtained slopes of 0.31 g of urine N, excreted per g of N ingested, and 0.21 g of fecal N, excreted per g of N ingested, showing the coefficients of determination values (r^2^) of 0.75 and 0.81, respectively. The authors of [94] calculated slope values of 0.12 g of urine N excreted per g of N ingested and 0.45 g of fecal N excreted per g of N ingested, as well as similar values for the coefficients of determination (0.75 and 0.81, respectively). In both cases, these data were quite similar to those given in Table 5. Zhao et al. [94] reported average N intakes of 24.6 g and average N excretions of 6.4 g of fecal N and 12.6 g of urine N.

Estimations of urine and fecal N excretion were estimated from N intake by using the equations shown in Table 5. The calculations were quite similar and were equivalent to 6.3 g of fecal N and to 15.5 g of urine N. Similarly, urine output, fecal N excretion, and urine N excretion were compared to the results by Beverley et al. [95] from sheep grazing annual ryegrass (63.9% OMD), with an average daily intake of 52.4 g of N per sheep. The results showed an average urine output of 3082 mL and the excretion of 13.8 g of fecal N and 35.4 g of urine N, compared to a urine output of 4550 mL and the excretion of 4.43 g of fecal N and of urine N, calculated for an average daily intake of 21 g of N per sheep and a similar OMD rate (63%).

### 4.2. Forage Production Model

Mushataq et al. [96] pointed out that plant height was the best genotypically related variable to biomass production (r = 0.56); in any case, it was lower than the calculation from the present work (0.93). However, Bilal et al. [97] reported outputs of 87 kg of DM per kg of N fertilizer and 0.21 cm of plant height per kg of N fertilizer, applied to oat crops, and higher than 62.9 kg of DM per kilogram of N fertilizer applied as a basal dressing. DM production in both cereal crops (triticale and oat) was 5.9 and 5.2 tonnes per hectare, respectively, and the estimated production was 5.99 and 5.4 tonnes per hectare, respectively. According to data from the Spanish Ministry of Agriculture, Fisheries and Food [98], the average performance of cereal grain crops that were used as forages was 3.9 and 3.4 tonnes of DM per hectare in Spain and Castilla-La Mancha region, respectively, assuming 35% of DM content at harvest. These data could be in line with the estimations shown in Table 5.

### 4.3. Mitigation Strategies

The main strategies for mitigation of the effects of GHG emissions in sheep farms are based on improving their productivity by enhancing different factors related to fertility, longevity, animal feed efficiency [99,100], and feedstuffs production [101]. Improving efficiency has been identified as the most relevant mitigation option [102]. Genetics, farm management, or feeding management, among others, could contribute to increasing efficiency at the farm level [103], by reducing the number of animals without cutting the production down. Genetics could lead to a reduction in the emissions at the flock level, but inheritability estimations related to individual CH_4_ production are low [104].

Increasing the genetic value in the Manchega breed as a whole, by up to 5%, 10%, or 15%, could lead to a reduction in the number of adult animals of between 0.47% and 2.38% without negatively affecting milk production. This reduction in the number of animals could cause a reduction in GHG emissions of between 0.16% and 1.78% CO_2_e per hectare.

Many other management approaches, such as a reduction in replacement rates, reduction from lambs’ deaths or lactating females’ deaths, or the reduction in the culling rates of non-productive females, could reduce the GHG emissions in terms of CO_2_e per hectare and per liter of milk by between 0.93 and 1.35% in M, 0.32 and 0.53% in F, and 1.15 and 2.49% in FL (Table 5). Cruickshank et al. [105] reported a drop in the enteric methane emissions that was equivalent to 0.04% when reducing mortality rates down to 10% in adult sheep and 1.3% in the case of lambs. In this study, a theoretical scenario with a reduction of 5% in adult animals’ mortality, combined with a reduction of 5% in lambs’ mortality, led to reductions in emissions per breeding female that varied from 0.13% to 0.44% in M, 0.02% to 0.47% in F, and 0.86% to 2.2% in FL (data not shown) [106].

Changes in feed management are considered to be the most variable mitigation strategy (Table 5). The replacement of soybean with peas reduced the carbon footprint by up to 14.9% in M and up to 22.2% in FL, as a consequence of the reduction in the emissions coming from imports [77] and from indirect land-use change (iLUC) [107].

Concentrates based on by-products increased the GHG emissions by an average of 20.3% in all cases, due to the higher production of enteric CH_4_ as a consequence of higher fiber levels in the animals’ diets [108]. Nevertheless, this kind of concentrate could also lead to an increase in the emissions linked to land-use changes [107], derived from their lower nutritional value, which, in turn, could lead to higher inputs at farm level (Table 5). 

Assuming an emission factor of 9.73 kg CO_2_e per kg of formula [109], the use of formula as a replacement for breastmilk in lambs and kids feeding could increase the carbon footprint per liter of marketed milk up to 17.7% in M, 11.7% in F, and 13.1% in FL.

Ruminants have the ability to transform high-fiber feedstuffs into meat and milk without competing with humans [109]. In the case of the Manchega breed, grazing triticale on-farm reduced emissions by up to 4.07% per hectare and 1.13% per liter of milk, as a consequence of reducing 4.5% CH_4_ emissions per hectare and increasing 2.34% carbon sequestration per hectare.

Similar to [110], replacing structural carbohydrates (cellulose and hemicellulose) with non-structural carbohydrates (starch and sugars) would reduce the production of enteric CH_4_. Conversely, reducing feedstuffs by 15% through grazing triticale grasslands in springtime could ensure a 2.07% reduction in CH_4_ emissions per hectare (Table 5).

In the Manchega breed, replacing poorly digestible feedstuffs with more digestible oat hay led up to a 3.4% reduction in emissions per hectare and up to a 1.13% reduction in emissions per liter of milk. In the case of the foreigners’ breeds, those reductions were 0.43% and 0.43%, respectively, and, in the case of the Florida goats, those reductions reached 6.6% and 1.11%, respectively.

Negative effects on milk production in the Manchega breed, linked to an ambient temperature rise, were studied by [92]. The results indicated an average comfort temperature zone varying from 11 °C to 21 °C; from 19 °C to 30 °C was a maximum temperature for those animals with a higher milk performance. In this study, the loss in milk production was 0.46%, which is within the range noted by [91] in the Manchega breed (from 0.1% to 2.6%). This decrease in milk production led to an increase in emissions per liter of milk, up to 0.95% in M, 1.3% in F, and 0.72% in FL (Table 5).

## 5. Conclusions

The ManleCO_2_ model has been described and employed for simulating different strategies aiming to mitigate the GHG emissions effects in three ruminant breed groups, a Spanish autochthonous sheep breed (Manchega), two foreign sheep breeds (Lacaune and Assaf), and a Spanish autochthonous goat breed (Florida). The carbon footprints obtained in this study were similar to others described in the scientific literature for both sheep and goats, regardless of the breed, varying from 2.01 to 5.62 kg CO_2_e per liter of fat- and protein-corrected milk (FPCM). This work revealed that foreign breed small ruminant flocks are much more efficient in terms of carbon footprint per liter of milk than those made up of Spanish autochthonous-breed small ruminants. However, when comparing carbon footprint per hectare, flocks made up of Spanish autochthonous-breed small ruminants are the most efficient. The different scenarios that were analyzed in this study showed that feedstuff purchase is the most relevant variable affecting the increase in GHG emissions per hectare, per livestock unit, and per liter of fat- and protein-corrected milk (FPCM). In fact, its use increased GHG emissions by up to 3.84% in all the flocks considered as a whole. Simulated strategies related to animal feeding had a higher impact on GHG emissions per liter of FPCM. Among these strategies, we would highlight that the replacement of soybean with peas in the concentrates could reduce GHG emissions by 18.7%, but they could be increased by up to 20.3% when replacing conventional concentrates with others based on by-products. On the other hand, the substitution of milk with milk replacements in suckling lambs could increase GHG emissions by up to 14.2% per liter of FPMC. A future study, including different goat breeds, such as the Murciano-Granadina or a larger number of Lacaune and Assaf sheep flocks, could help to consolidate the results obtained in this work.

## Figures and Tables

**Table 1:** Characteristics of the herds used in the simulation.

Sources of Variation (Baseline)	Manchega	Foreigners	Florida
Total, ha	1013	157	218
Animals	1466	1466	228
Lactating animals	838	1197	122
Non-lactating animals	634	248	106
Replacement animals	414	489	52
Milk, liters or FPCM per head and year	282	497	467
Purchased fodder, kg ha^−1^	450	4120	238
Purchased concentrates, kg ha^−1^	533	7147	484
Grazing occupation, %	17	3	11
Feeding stuffs	OH; AlH,CS,Ba,Con	OH; AlH,CS,Ba,Con	OH; AlH,CS,Ba,Con
Fertilizers, kg of N per ha	24.6	27.7	11.1
Fertilizers, kg of P per ha	7.6	7.7	0.13
Fertilizers, kg of K per ha	5.2	7.6	0.13
CO_2_e, kg per ha^−1^ and per year	1655	12,634	1198
CO_2_e, kg per LU and per year	6397	7510	6507
CO_2_e, kg per liter of FPCM	3.78	2.77	3.06

OH: oat hay; AlH: alfalfa hay; CS: cereal straw; Ba: barley; Con: concentrates; LU: livestock unit.

**Table 2:** Simulated scenarios.

Breed	Group	Scenario
Manchega	Genetic improvement	5% genetic value
		10% genetic value
		15% genetic value
Manchega Foreigners Florida	Animals inventory	<10% unproductive females
		<5% replacement
		<5% dead offspring
		<5% deaths of lactating animals
Manchega Foreigners Florida	Purchased Feed	Soybean replacement by peas in food
		Replacement of feedstuffs by fibrous ones
		Natural breastfeeding × automatic breastfeeding
Manchega Foreigners	Forage Management	Substitution of 25% land oat (silage round bale) × vetch
		Substitution of 25% land oat (silage bags) × vetch
		Triticale grazing 100 days ^A^
		<15% of fodder grains and triticale grazing ^A^
		Substitution of oat hay (113 RFQ vs. 139)
Manchega Foreigners Florida	Electrical supply	Reduce 10% milking energy
Manchega	Climate change	Temperature increase + 2 °C

^A^: not in goats; RFQ: relative forage quality [87].

**Table 3:** Technical and productive features at farm level (*n* = 36 farms).

Sources of Variation	Manchega (M = 25) (Value (sd))	Foreigners (F = 6) (Value (sd))	Florida (C = 5) (Value (sd))
Land			
Total, n◦ has	1013 (814)	157 (215)	218 (339)
Arable, n◦ has	164 (168)	78 (88)	9 (7)
Fallow land, n◦ has	67 (93)	27 (48)	2 (2)
Agricultural cereals (G), n◦ has	40 (51)	28 (57)	4 (3)
Agricultural cereals (F), n◦ has	35 (40)	38 (50)	4 (6)
Maize, n◦ has	6 (22.7)	-	-
Legumes, n◦ has	16 (23.8)	-	-
Communal pastures, n◦ has	849 (843)	79 (159)	209 (338)
Forages and grains production per farmland (hectare)			
Alfalfa, t DM ha^−1^	14.7 (3.6)	-	-
Maize, t DM ha^−1^	15.7 (2.8)	-	-
Oat, t DM ha^−1^	4.9 0.9)	5.0 (0.3)	5.1 (0.3)
Triticale, t DM ha^−1^	4.6 (0.3)	5.1 (0.1)	-
Vetch, t DM ha^−1^	5.2 (0.3)	-	-
Peas, t DM ha^−1^	3.7 (2.0)	-	-
Barley grain, t DM ha^−1^	2.9 (0.7)	3.0 (0.1)	2.5 (0.2)
Fertilizers per farmland (hectare)			
Fertilizers, kg N ha^−1^	24.6 (37.2)	27.7 (42.7)	11.1 (17.5)
Fertilizers, kg P ha^−1^	7.6 (25.7)	7.7 (11.8)	0.13 (0.21)
Fertilizers, kg K ha^−1^	5.2 (14.9)	7.6 (11.8)	0.13 (0.21)
Animals			
Total, n	1466 (980)	1446 (1162)	228 (85)
Lactating female, n	838 (558)	1199 (950)	122 (42)
Flock Replacement, n	414 (326)	489 (470)	52 (29)
Flock Replacement, %	26.9 (7.7)	30.6 (6.9)	21.6 (5.5)
Stocking Density, LU ha^−1^	1.12 (2.1)	129.7 (170)	9.4 (15,4)
Feed			
Ingested, kg DM PF year^−1^	1025 (141)	1066 (166)	790 (51)
Purchased forage, kg DM PF year^−1^	236 (119)	338 (90)	250 (148)
Purchased concentrate, kg DM PF year^−1^	304 (44)	586 (143)	430 (59)
Own forage, kg DM PF year^−1^	449 (203)	93 (114)	109 (120)
Own concentrate, kg DM PF year^−1^	36 (44)	49 (88)	.
Grazing time per year, %	27.9 (14.1)	3.7 (7.1)	16.5 (17.1)
Meat and milk yield			
Milk FPCM, t farm ^A^	393.7 (234)	691.8 (632)	79.6 (21.8)
Milk FPCM, t ha^−1 B^	1.5 (2.6)	285.4 (371)	17.4 (29.4)
Milk FPCM, liters per PF ^1 B^	307 (76)	479 (94)	381 (63)
Offspring born, ha	9 (18.1)	1021 (1352)	57 (105)
Offspring slaughtered for meat, ha ^C^	4.6 (9.5)	443 (559)	33.6 (52)
Cull animals, ha	1.1 (2.4)	121 (164)	11.8 (19)
Live weight sold, kg ha year^−1^	85.7 (180)	8664 (11,072)	671 (1057)
Efficiency			
LU	4.7 (1.8)	4.5 (1.9)	3 (0)
Marketed milk FPCM, t LU^−1^	80.5 (30.7)	131.9 (69.8)	26.5 (7.3)
Cheese extract, t LU^−1^	11.5 (4.1)	16.5 (9.5)	2.3 (0.7)
Live weight sold, t LU^−1^	4.9 (1.6)	4.8 (1.3)	1.1 (0.7)
Liters FPCM kg^−1^ DM ingested ^D^	0.30 (0.07)	0.46 (0.13)	0.48 (0.06)
Liters FPCM kg^−1^ DM milking ^E^	0.60 (0.20)	0.72 (0.20)	0.78 (0.15)
NUE _farm,_ %	22.5 (10.8)	16.3 (3.8)	25.9 (15.3)
NUE _milk-lactating females,_ %	20.3 (6.3)	15.5 (3.9)	21.5 (7.6)
NUE _milk+meat all animals together_ %	33.5 (7.5)	29.3 (4.8)	34.1 (9.0)

sd: standard deviation; G: grain; F: forage; DM: dry matter; PF: present female; FPCM: fat- and protein-corrected milk; ^A^: marketed milk; ^B^: marketed milk + milk consumed by offspring; ^C^: slaughtered offspring for meat production + breeding animals + discards; LU: agricultural work unit; NUE: nitrogen use efficiency; PUE: phosphorous use efficiency; ^D^: group of animals; ^E^: lactating female.

**Table 4:** Main characteristics of the animal model, the excreta production model (urine and fecal N), and the forage production model.

**Animal Model**	**Data Set**		**Characteristics of the Independent Variables**				
			**Non-Standardized** **Coefficients**		**Standardized** **Coefficients**	**Collinearity** **Diagnosis**	
Independent variables	Mean	sd	β	se	β	Tol	VIF
Replacement females (4–12 months)							
Constant			−136.7 **	61.6			
Present Female	1267	873	0.42 ***	0.04	0.809	1	1
Born lambs and kids							
Constant			−112.1 NS	89.1			
Present Female	1267	873	1.91 ***	0.058	0.974	1	1
Culled lambs and kids							
Constant			91.4 NS	75.8			
Present Female	1267	873	0.98 ***	0.049	0.936	1	1
Losses, deaths, discards (breeding ewes/goats)							
Constant			−122.6 NS	74.0			
Present Female	1267	873	0.4 ***	0.04	0.904	1	1
Lambs and kids deaths, abortions, etc.							
Constant			−117.2 ^NS^	101.2		
Present Female	1267	873	0.55 ***	0.068	0.768	1	1
**Feces, Urine and N Excretion Model (per Head and Day)**	**Data Set**		**Characteristics of the Independent Variables**				
			**Non-Standardized** **Coefficients**		**Standardized** **Coefficients**	**Collinearity** **Diagnosis**	
Independent variables	Mean	sd	β	se	β	Tol	VIF
Faeces, g d^−1^							
Constant			512.7 ***	18.5		
OMD	0.633	0.10	−699.1 ***	27.3	−0.726	0.905	1.1
DM intake, g sheep d^−1^	791.5	186	0.084 *	0.043	0.160	0.100	9.2
GP intake, g sheep d^−1^	143.8	60.6	0.579 ***	0.083	0.357	0.279	3.59
NDF intake, g sheep d^−1^	386.7	104.9	0.269 ***	0.067	0.288	0.143	7.00
Model volume urine, cc d^−1^							
Constant			−654.1 ***	74.7			
N intake, g d^−1^	20.9	8.5	71.3 ***	3.51	0.752	1.0	1.0
N feces, g d^−1^							
Constant			1.30 ***	0.149		
N intake, g d^−1^	20.9	8.5	0.241 ***	0.007	0.852	1.0	1.0
N urine, g d^−1^							
Constant			1.95 ***	0.482		
N intake, g d^−1^	20.9	8.5	0.64 ***	0.021	0.804	1.0	1.0
**Forage Production Model**	**Data Set**		**Characteristics of the Independent Variables**				
			**Non-Standardized** **Coefficients**		**Standardized** **Coefficients**	**Collinearity** **Diagnosis**	
Independent variables	Mean	sd	β	se	β	Tol	VIF
Triticale, kg DM ha^−1^							
Constant			−11,052 ***	833		
Height, cm	82.4	18.22	133.2 ***	4.86	0.926	0.97	1.02
Days to inflorescence emergence	154.2	36.5	33.9 ***	3.56	0.471	0.45	2.19
kg N ha^−1^ background	23.8	14.1	62.9 ***	9.21	0.339	0.45	2.20
Oats, kg DM ha^−1^							
Constant			2632 ***	561.7		
Height, cm	70.5	28.0	112.6 ***	7.4	0.935	1.0	1.0

OMD: Organic matter digestibility; DM: dry matter; GP: gross protein; NDF: neutral detergent fiber; sd: standard deviation; se: standard error; Tol: tolerance; VIF: variance inflation factor; NS: non-significant; * significance is considered for *p* < 0.05; ** *p* < 0.01; *** *p* < 0.001.

**Table 5:** Statistical evaluation of the models for the estimation of the number of animals and manure and forage production.

	Model	n	se	R^2^	D-W	Observed	Simulated	d	R^2^	RMSE	MBE	EF
**Animal**												
Replacement females (4–12 months)	−136.7 + (0.42 PF)	61	271	0.65	2.08	400	406	0.98	0.93	1.88	−0.86	0.93
Born lambs and kids	−112.1 + (1.91 PF)	61	392	0.95	1.89	2343	2238	0.99	0.97	4.72	2.43	0.97
Culled lambs and kids	91.4 + (0.97 PF)	61	326	0.87	2.18	1329	1355	0.98	0.92	4.26	1.06	0.91
Losses, deaths, discards (breeding ewes/goats)	122.6 + (0.40 PF)	19	189	0.80	1.54	284	329	0.98	0.98	3.45	−3.1	0.92
Lambs’ and kids’ deaths, abortions, etc.	−117.2 + (0.55 PF)	48	377	0.59	1.84	578	565	0.96	0.92	4.58	1.06	0.85
Lambs and kids live weight on slaughter ^A-B^	$y=\beta_{1}e^{-\beta_{2e^{-\beta 3t}}}$	272	-	0.95	-	50.1	48.4	0.99	0.99	0.0079	−5.69	0.99
**Urine and Fecal N Excretion per Head and per Day**												
Feces, g DM ^C-D^	523-(692.6 OMD/100) + (0.084 g DM ingested d^−1^) + (0.57 g GP d^−1^) +(0.269 * g NDF d^−1^)	510	59.3	0.64	1.31	322.5	320.4	0.87	0.63	0.11	3.26	0.63
Urine, ml ^C-D^	−654.1 + (71.3 g N d^−1^)	313	529	0.56	0.85	757	751	0.84	0.74	1.7	2.43	0.66
N faeces, g ^C-D^	1.30 + (0.24 g N d^−1^)	510	1.26	0.78	0.99	6.38	6.35	0.97	0.72	0.0025	1.98	0.99
N urine, g^C-D^	1.95 + (0.64 g N d^−1^)	313	4.06	0.64	0.75	15.6	15.4	0.87	0.64	0.008	−5.6	0.90
N feces, g ^E^	0.16 + (0.3 g N ingested kg live weight^0.75^)		0.065	0.91	-	-	-	-	-	-	-	-
N urine, g ^E^	−0.0061 + (0.31 g N ingested kg live weight^0.75^)		0.06	0.98	-	-	-	-	-	-	-	-
**Forage Production**												
Triticale, kg DM ha^−1^	−11,952 + (133.2 Height, cm) + (33.9 Days to inflorescence emergence ) + (62.9 kg N background)	59	669	0.93	1.67	5903	5990	0.98	0.65	8.46	0.63	0.52
Oats, kg DM ha^−1^	−2632 + (112 Height, cm)	35	1211	0.87	0.59	5311	5407	0.95	0.87	34.5	0.63	0.76

^A^: Sheep live weight up to 480 days of age for Manchega sheep (Gompertz model; t = age in days; β1 (68.59), β2 (2.47), β3 (0.01)) ^B^: Goats live weight up to 480 days of age (Gompertz model; t = age in days; β1 (53.3), β2 (1.9), β3 (0.0046)), according to [34]; PF: present female; se: standard error; R^2^: coefficient of determination; ^C^: lactating females; ^D^: non-lactating females; ^E^: replacement animals [36]; N: nitrogen; DM: dry matter; OMD: organic matter digestibility); GP: gross protein; NDF: neutral detergent fiber; se: standard error; et: estimation error; D–W: Durbin–Watson; d: index of agreement; RMSE: root mean square error; MBE: mean bias error; EF: model efficiency.

**Table 6:** Potential change in the carbon footprint of sheep and goat farms in Castilla-La Mancha, linked to changes in management model.

Breed	Group	Scenario	CO_2_e kg/ha	Change%	CO_2_e kg/LU	Change %	CO_2_e kg/liter FPCM	Change %
Manchega	Baseline	−	1655		6397		3.78	
Foreigners		−	12,634		7510		2.77	
Florida		−	1198		6507		3.06	
		5% genetic value	1652	−0.17	6387	−0.17	3.77	−0.17
Manchega	Genetics	10% genetic value	1631	−1.45	6356	−0.65	3.73	−1.37
		15% genetic value	1625	−1.81	6411	0.21	3.72	−1.60
Manchega	Animal inventory	< 5% replacement	1454	−12.1	5620	−12.1	3.78	−0.17
		< 5% offspring deaths	1656	0.03	6399	0.03	3.76	−0.59
		< 5% lactating animals deaths	1454	−12.1	5620	−12.1	3.76	−0.56
		< 10% empty females	1616	−2.38	6456	0.92	3.71	−2.05
Foreigners		< 5% replacement	11,357	−10.1	6751	−10.1	2.77	−0.11
		< 5% offspring deaths	12,637	0.02	7512	0.02	2.77	−0.33
		< 5% lactating animals deaths	11,408	−9.70	6706	−10.1	2.77	−0.21
		< 10% empty females	12,535	−0.78	7562	0.69	2.76	−0.69
Florida		< 5% replacement	1012	−15.5	5497	−15.5	3.07	0.20
		< 5% offspring deaths	1036	−13.5	5627	−13.5	3.07	0.29
		< 5% lactating animals deaths	1218	1.65	6539	0.50	3.07	0.38
		< 10% empty females	1164	−2.82	6599	1.42	2.98	−2.82
Manchega		Milk replacer	1775	7.25	6861	7.25	4.42	16.7
	Purchased feed	Soybean x peas	1435	−13.2	5548	−13.2	3.28	−13.2
		Conventional vs. fibrous feedstuffs	1862	12.5	7198	12.5	4.26	12.5
		Milk replacer	13,390	5.99	7960	5.99	3.08	11.1
Foreigners		Soybean x peas	10,471	−17.1	6225	−17.1	2.30	−17.1
		Conventional vs. fibrous feedstuffs	15,867	25.5	9432	25.5	3.48	25.5
		Milk replacer	1292	7.88	7019	7.88	3.44	12.2
Florida		Soybean x Peas	961	−19.7	5219	−19.7	2.46	−19.7
		Conventional vs. fibrous feedstuffs	1430	19.3	7768	19.3	3.66	19.3
Manchega	Forage Management	Oat hay RFV 113 vs. 139	1427	−13.7	5516	−13.7	3.78	−0.08
		Grazing triticale 100 days	1417	−14.3	5477	−14.4	3.80	0.39
		< 15% high-protein feedstuffs and triticale grass	1632	−1.41	6307	−1.41	3.73	−1.41
		< 25% aurface V-O (hay x bag silage) and vetch	1621	−2.04	6267	−2.04	3.71	−2.04
		< 25% surface V-O (hay x silage round bales) and vetch	1655	0.00	6398	0.00	3.79	0.00
Foreigners		Oat hay RFV 113 vs. 139	12,565	−0.55	7469	−0.55	2.76	−0.55
		< 25% surface V-O (hay x bag silage) and Vetch	10,471	−17.1	6225	−17.1	2.30	−17.1
		< 25% surface V-O (hay x silage round bales) and vetch	15,867	25.5	9732	25.5	3.48	25.5
Florida		Oat hay RFV 113 vs. 139	1001	−16.4	5437	−16.4	3.08	0.45
Manchega	Electrical supply		1653	−0.15	6388	−0.15	3.78	−0.15
Foreigners		< 10% milking time	12,622	−0.10	7503	−0.10	2.77	−0.10
Florida			1012	−15.5	5497	−15.5	3.07	0.11
Manchega	Room temperature increase	+2.0 °C	1659	0.22	6412	0.22	3.84	1.46
Foreigners			12,754	0.95	7581	0.95	2.79	0.51
Florida			1201	0.28	6525	0.28	3.08	0.70

FPCM: Fat- and protein-corrected milk; RFV: Relative forage value; V-O: vetch-oat.

## Data Availability

Data supporting reported results are not archived in any public access repository. Nevertheless, if access were needed, please send your requirement to rgcano@jccm.es and we would kindly provide them.

## References

[1] Food and Agriculture Organization of the United Nations (FAO) (2016). Statistical Yearbook.

[2] Ministerio de Agricultura y Pesca, y Alimentación (MAPA) (2016). Resultados técnico-económicos del Ganado Ovino de leche en 2016, Subdirección General de Análisis, Prospectiva y Coordinación. Subsecretaría. https://www.mapa.gob.es/es/ministerio/servicios/analisis-y-prospectiva/ganadoovinodeleche_tcm30-520800.pdf.

[3] Ministerio de Agricultura y Pesca, y Alimentación [MAPA] (2020). Indicadores cuatrimestrales situación sector ovino leche España. Subdirección General de Producciones Ganaderas y Cinegéticas, Dirección General de Producciones y Mercados Agrarios. https://www.mapa.gob.es/es/ganaderia/estadisticas/ovinodeleche_indicadorsemestral_junio2021_rev_tcm30-428244.pdf.

[4] Montoro V., Vicente J., Rincón E., Pérez-Guzmán M.D., Gallego R., Rodríguez J.M., Arias R., Garde J.J. Actualidad de la producción de ovino lechero en la Comarca Montes Norte de Ciudad Real: I. Estructura de las explotaciones. Proceedings of the XXXII Jornadas Científicas y XI Jornadas Internacionales de Ovinotecnia y Caprinotecnia.

[5] Toro-Mújica P., García A., Gómez-Castro A., Perea J., Rodríguez-Estévez V., Angón E., Barba C. (2012). Organic dairy sheep farms in south-central Spain: Typologies according to livestock management and economic variables. Small Rum. Res..

[6] De Vries M., De Boer I.J.M. (2010). Comparing environmental impacts for livestock products: A review of life cycle assessments. Livest. Sci..

[7] Gerber P.J., Steinfeld H., Henderson B., Mottet A., Opio C., Dijkman J., Falcucci A., Tempio G. (2013). Tackling Climate Change Through Livestock: A Global Assessment of Emissions and Mitigation Opportunities.

[8] Heilig G. (1994). The Greenhouse Gas Methane (CH_4_): Sources and Sinks, the Impact of Population Growth, Possible Interventions. Popul. Environ..

[9] Hristov A.N., Oh J., Lee C., Meinen R., Montes F., Ott T., Firkins J., Rotz A., Dell C., Adesogan A. (2013). Mitigation of Greenhouse Gas Emissions from Livestock Production: A Review of Technical Options for Non-CO_2_ Emissions.

[10] Smith P., Martino D., Cai Z., Gwary D., Janzen H., Kumar P., McCarl B., Ogle S., O’Mara F., Rice C. (2008). Greenhouse gas mitigation in agriculture. Philos. Trans. R. Soc. B Biol. Sci..

[11] Fraser M.D., Fleming H.R., Theobald V.J., Moorby J.M. (2015). Effect of breed and pasture type on Methane emissions from weaned lambs offered fresh forage. J. Agric. Sci..

[12] Zhao Y.G., Aubry A., O’Connell N.E., Annett R., Yan T. (2015). Effects of breed, sex and concentrate supplementation on digestibility, enteric Methane emissions, and nitrogen utilization efficiency in growing lambs offered fresh grass. J. Anim. Sci..

[13] Sanjo V.S., Sejian V., Bagath M., Ratnakaran A.P., Lees A.M., Al-Hosni Y.A.S., Sullivan M., Bhatta R., Gaughan J.B. (2016). Modelización de emisiones de gases de efecto invernadero procedentes del ganado. Front. Environ. Sci..

[14] Lesschen J.P., van den Berg M., Westhoekb H.J., Witzkec H.P., Oenema O. (2011). Greenhouse gas emission profiles of European livestock sectors. Anim. Feed Sci. Technol..

[15] Kram T., Stehfest E., Bouwman A.F., Kram T., Goldewijk K.K. (2006). The IMAGE model: History, current status and prospects. Integrated Modelling of Global Environmental Change.

[16] Olesen J.E., Schelde K., Weiske A., Weisbjerg M.R., Asman W.A.H., Djurhuus J. (2006). Modelling greenhouse gas emissions from European conventional and organic dairy farms. Agric. Ecosyst. Environ..

[17] Schils R.L.M., De Haan M.H.A., Hemmer J.G.A., Van den Pol-van Dasselaar A., De Boer J.A., Evers A.G., Holshof G., Van Middelkoop J.C. (2007). DairyWise, a whole-farm dairy model. J. Dairy Sci..

[18] Del Prado A., Misselbrook T., Chadwick D., Hopkins A., Dewhurst R.J., Davison P. (2011). SIMSDAIRY: A modelling framework to identify sustainable dairy farms in the UK. Framework description and test for organic systems and N fertilizer optimization. Sci. Total Environ..

[19] Saletes S., Fiorelli J., Vuichard N., Cambou J., Olesen J.E., Hacala S., Sutton M., Fuhrer J., Soussana J.F., Kaltschmitt M., Weiske A. (2004). Greenhouse gas balance of cattle breeding farms and assessment of mitigation options. Greenhouse Gas Emissions from Agriculture. Mitigation Options and Strategies.

[20] Chianese D.S., Rotz C.A., Richard T.L. (2009). Whole-farm gas emissions: A review with application to a Pennsylvania dairy farm. Appl. Eng. Agric..

[21] Hutchings N.J., Kristensen I.B. (2016). Measures to reduce the greenhouse gas emissions from dairy farming and their effect on nitrogen flow. Abstracts 19th N Workshop, Proceedings of Efficient Use of Different Sources of Nitrogen in Agriculture–From Theory to Practice, Skara, Sweden, 27 June–29 June 2016.

[22] Salcedo G., Salcedo-Rodríguez D. (2021). Valoración holística de la sostenibilidad en los sistemas lecheros de la España húmeda. ITEA-Inf. Técnica Económica Agrar..

[23] Neumann K., Verburg P.H., Elbersen B., Stehfest E., Woltjer G.B. (2011). Multi-scale scenarios of spatial-temporal dynamics in the European livestock sector. Agric. Ecosyst. Environ..

[24] Bell M.J., Eckard R.J., Cullen B.R. (2012). The effect of future climate scenarios on the balance between productivity and greenhouse gas emissions from sheep grazing systems. Livest. Sci..

[25] Bohan A., Shalloo L., Malcolm B., Ho C.K.M., Creighton P., Boland T.M., McHugh N. (2016). Description and validation of the Teagasc Lamb Production Model. Agric. Syst..

[26] Pulina G., Macciotta N., Nudda A. (2005). Milk composition and feeding in the Italian dairy sheep. Ital. J. Anim. Sci..

[27] Food and Agriculture Organization (FAO) (2009). CROPWAT 8.0 Model.

[28] McMaster G., Wilhelm W. (1987). Growing degree-days: One equation, two interpretations. Agric. For. Meteorol..

[29] Belsley D. (1991). Conditioning Diagnostics: Collinearity and Weak Data in Regression.

[30] Willmott C.J. (1982). Some comments on the evaluation of model performance. Bull. Am. Meteorol. Soc..

[31] Nash J.E., Sutcliffe J.V. (1970). River flow forecasting through conceptual models part I—A discussion of principles. J. Hydrol..

[32] Ministerio de Agricultura y Pesca, y Alimentación (MAPA) Real Decreto 1131/2010, de 10 de septiembre, por el que se establecen los criterios para el establecimiento de las zonas remotas a efectos de eliminación de ciertos subproductos animales no destinados a consumo humano generados en las explotaciones ganaderas. (BOE 2-10-2010). https://www.boe.es/diario_boe/txt.php?id=BOE-A-2010-15123.

[33] Regadas J.G., Tedeschi L.O., Rodrigues M.T., Brito L.F., Oliveira T.S. (2014). Comparison of growth curves of two genotypes of dairy goats using nonlinear mixed models. J. Agric. Sci..

[34] Vega C. (2000). La relación paja-grano en los cereals. (Una aproximación en condiciones de secano semiárido, en Aragón). Inf. Técnicas Dep. Agric. Medio Ambiente.

[35] Allen R.G., Pereira L.S., Raes D., Smith M. (1998). Crop Evapotranspiration Guidelines for Computing Crop Water Requirements-FAO Irrigation and Drainage Paper 56.

[36] Salcedo G. (2020). Emisiones en la producción de forrajes de las explotaciones lecheras. ITEA Inf. Técnico Económica Agrar..

[37] Cela S., Santiveri F., Lloveras J. (2013). Short communication. Nitrogen content of residual alfalfa taproots under irrigation. Span. J. Agric. Res..

[38] Petersen B.M., Knudsen M.T., Hermansen J.E., Halberg N. (2013). An approach to include soil carbon changes in life cycle assessments. J. Clean. Prod..

[39] Chirinda N., Olesen J.E., Porter J.R. (2012). Root carbon input in organic and inorganic fertilizer-based systems. Plant Soil.

[40] Escudero A., Gonzalez-Arias A., del Hierro O., Pinto M., Gartzia-Bengoetxea N. (2012). Nitrogen dynamics in soil amended with manures composted in dynamic and static systems. J. Environ. Manag..

[41] Rodríguez A. (2013). Análisis de la Rentabilidad en las Explotaciones de Ovino de Leche en Castilla y León. Ph.D. Thesis.

[42] Bodas R., Tabernero de Paz M.J., Bartolomé D.J., Posado R., García J.J., Olmedo S., Rodríguez L. (2013). Consumo eléctrico en granjas de ganado ovino lechero de Castilla y León. Arch. Zootec..

[43] de Blas C., Mateos G.G., García-Rebollar P., FEDNA (2010). Tablas de Composición y Valor Nutritivo de Alimentos Para la Fabricación de Piensos Compuestos.

[44] Alderman G., Cottrill B.R. (1993). Energy and Protein Requirements of Ruminants: An Advisory Manual Prepared by the AFRC Technical Committee on Responses to Nutrients. https://agris.fao.org/agris-search/search.do?recordID=GB9406276.

[45] Ministry of Agriculture, Fisheries and Food (1990). UK Tables of Nutritive Value and Chemical Composition of Feeding Stuffs.

[46] National Research Council (NRC) (2001). Nutrient Requirements for Dairy Cattle.

[47] Vermorel M., Coulon J.B., Journet M. (1987). Révision du systéme des unités fourragères (UF). Bull. Tech. Cent. Rech. Zootech. Vétérinaires Theix.

[48] National Research Council (NRC) (1985). Nutrient Requirements of Sheep.

[49] Tedeschi L.O., Molle G., Menendez H.M., Cannas A., Fonseca M.A. (2019). The assessment of supplementation requirements of grazing ruminants using nutrition models. Transl. Anim. Sci..

[50] Pulina G., Bettati T., Serra F.A., Cannas A. (1996). Razi-O: Development and validation of a software for dairy sheep feeding. Proceedings of the XII National Meeting of the Societa Italiana di Patologia e d’Allevamento Degli Ovini e dei Caprini.

[51] Institut National de la Recherche Agronomique (INRA) (2007). Alimentation des Bovins, Ovins et Caprins: Besoins des Animaux—Valeurs des Aliments—Tables Inra.

[52] Macoon B., Sollenberger L.E., Moore J.E., Staples C.R., Fike J.H., Portier K.M. (2003). Comparison of three techniques for estimating the forage intake of lactating dairy cows on pasture. J. Anim. Sci..

[53] Freer M. (2007). Nutrient Requirements of Domestical Ruminants.

[54] Molina M.P., Caja G., Torres A., Gallego L. (1991). ITEA: Producción Animal. Inf. Técnica Económica Agrar..

[55] Bocquier F., Barillet F., Guillouet P. (1991). Prediction of gross energy content of ewe’s milk from different chemical analysis: Proposal of an energy corrected milk for dairy ewes. Energy Metabolism of Farm Animals.

[56] Aguilera J.F., Prieto C., Fonollá J. (1990). Protein and energy metabolism of lactating Granadina goats. Brit. J. Nutr..

[57] Cannas A., Tedeschi L.O., Fox D.G., Pell A.N., van Soest P.J. (2004). A mechanistic model for predicting the nutrient requirements and feed biological values for sheep. J. Anim. Sci..

[58] Institut National de la Recherche Agronomique (INRA) (1989). Alimentación de los Rumiantes.

[59] Brentrup F., Küsters J., Lammel J., Kuhlmann H. (2000). Methods to estimate on-field nitrogen emissions from crop production as an input to LCA studies in the agricultural sector. Intern. J. Life Cycle Assess..

[60] Christelle R., Pflimlin A., Le Gall A. Optimisation of environmental practices in a network of dairy farms of the Atlantic Area. Proceedings of the Final Seminar of the Green Dairy Project.

[61] NRC (2007). Nutrient Requirements of Small Ruminants: Sheep, Goats, Cervids and World Camelids.

[62] Agricultural Research Council (ARC) (1980). The Nutrient Requirements of Ruminant Livestock.

[63] Ogejo J.A., Wildeus S., Knight P., Wilke R.B. (2020). Estimating goat and sheep manure production and their nutrient contribution in the Chesapeake Bay Watershed. Appl. Eng. Agric..

[64] Del Prado Santeodoro A., Baucells Ribas J., Casasús Pueyo I., Fondevila Camps M. (2019). Bases Zootécnicas Para el Cálculo del Balance Alimentario de Nitrógeno y Fósforo en Ovino.

[65] IPCC (2006). Guidelines for National Greenhouse Gas Inventories.

[66] De Vries J.W., Hoeksma P., Groenestein C.M. (2011). Life Cycle Assessment (LCA) mineral concentrates pilot. Wagening. UR Livest. Res..

[67] Goossensen F.R., Van Den Ham A. (1992). Equations to calculate nitrate leaching. Publicatie No. 33.

[68] Schils R., Oudendag D., van der Hoek K., de Boer J., Evers A., de Haan M. (2006). Broeikasgas Module BBPR.

[69] Velthof G.L., Mosquera J. (2011). Calculations of Nitrous Oxide Emissions from Agriculture in The Netherlands: Update of Emission Factors and Leaching Fraction.

[70] Velthof G., Oenema O. (1997). Nitrous oxide emission from dairy farming systems in the Netherlands. Neth. J. Agric. Sci..

[71] Thornthwaite C.W. (1948). An approach toward a rational classification of climate. Geogr. Rev..

[72] Kaspar H.F., Tiedje J.M. (1981). Dissimilatory reduction of nitrate and nitrite in the bovine rumen: Nitrous oxide production and effect of acetylene. Appl. Environ. Microb..

[73] Nielsen P.H., Nielsen A.M., Weidema B.P., Dalgaard R., Halberg N. (2003). LCA Food Data Base.

[74] Rotz C., Michael S., Chianese D., Montes F., Hafner S., Colette C. (2012). The Integrated Farm System Model.

[75] IDF (2010). A common carbon footprint approach for the dairy sector. The IDF guide to standard life cycle assessment methodology. Bull. Int. Dairy Fed..

[76] Audsley E., Brander M., Chatterton J., Murphy-Bokern D., Webster C., Williams A. (2009). How low can we go? An assessment of greenhouse gas emissions from the UK food System and the scope for to reduction them by 2050. Food Clim. Res. Netw..

[77] Battini F., Agostini A., Tabaglio V., Amaducci S. (2016). Environmental impacts of different dairy farming systems in the Po Valley. J. Clean. Prod..

[78] Chapagain A.K., Hoekstra A.Y. (2003). Virtual Water Flows between Nations in Relation to Trade in Livestock and Livestock Products.

[79] Chapagain A.K., Hoekstra A.Y. (2014). Water Footprints of Nations.

[80] Mekonnen M., Hoekstra A. (2012). A global assessment of the water footprint of farm animal products. Ecosystems.

[81] Thomson A.J., King J.A., Smith K.A., Tiffin D.H. (2007). Opportunities for Reducing Water Use in Agriculture.

[82] Bos J., de Haan J., Sukkel W., Schils R. (2014). Energy use and greenhouse gas emissions in organic and conventional farming systems in the Netherlands. NJAS Wagening. J. Life Sci..

[83] Ausdley E., Alber S., Clift R., Cowell S., Crettaz P., Gaillard G., Hausheer J., Jolliet O., Kleijn R., Mortensen B. (1997). Harmonization of Environmental Life Cycle Assessment for Agriculture. Final Report, Concerted Action AIR3-CT94-2028.

[84] Weidema B.P., Mortensen B., Nielsen P., Hauschild M. (1996). Elements of an Impact Assessment of Wheat Production. Inst. Prod. Dev..

[85] Sutton M.A., Billen G., Bleeker A., Erisman J.W., Grennfelt P., Grinsven H., Van Grizzetti B., Howard C.M., Leip A., Sutton M.A., Howard C.M., Erisman J.W., Billen G., Bleeker A., Grennfelt P., van Grinsven H., Grizzetti B. (2011). European Nitrogen Assessment—Technical summary. The European Nitrogen Assessment.

[86] Juárez M., Juárez M., Sánchez A., Jordá J.D., Sánchez J.J. (2004). Diagnóstico del Potencial Nutritivo del Suelo.

[87] Moore J.E., Undersander D.J. Relative forage quality: an alternative to relative value and quality index. Proceedings of the 13th Annual Florida Ruminant Nutrition Symposium.

[88] Legarra A., Ole F., Aguilar I., Misztal I. (2014). Single Step, a general approach for genomic selection. Livest. Sci..

[89] Wilkinson J.M., Davies D.R. (2012). The aerobic stability of silage: Key findings and recent development, Review paper. Grass Forage Sci..

[90] Salcedo G., Martínez-Suller L., Sarmiento M. (2009). Efectos del color de plástico y número de capas sobre la composición química y calidad fermentativa en ensilados de hierba y veza-avena. La Multifuncionalidad de los Pastos: Producción Ganadera Sostenible y Gestión de los Ecosistemas.

[91] Ramón M., Díaz C., Pérez-Guzman M.D., Carabaño M.J. (2016). Effect of exposure to adverse climatic conditions on production in Manchega dairy sheep. J. Dairy Sci..

[92] Finocchiaro R., van Kaam J.B., Portolano B., Misztal I. (2005). Effect of heat stress on production of Mediterranean dairy sheep. J. Dairy Sci..

[93] Patra A.K. (2010). Aspects of nitrogen metabolism in sheep-fed mixed diets containing tree and shrub foliages. Br. J. Nutr..

[94] Zhao Y.G., Gordon A.W., O´Connell N.E., Yan T. (2016). Nitrogen utilization efficiency and prediction of nitrogen excretion in sheep offered fresh perennial ryegrass (*Lolium perenne*). J. Anim. Sci..

[95] Beverley C., Ward K., Smith N., Gibbs G.J., Muir P. (2021). Effect of feeding time on urinary and faecal nitrogen excretion patterns in sheep. J. Agric. Res..

[96] Mushtaq A., Gul Z., Mir S.D., Dar Z.A., Dar S.H., Shahida I., Bukhari S.A., Khan G.H., Asima G. (2013). Estimation of correlation coefficient in oats (*Avena sativa* L.) for forage yield, grain yield and their contributing traits. Int. J. Plant Breed. Genet..

[97] Bilal M., Ayub M., Tariq M., Tahir M., Nadeem M.A. (2017). Dry matter yield and forage quality traits of oat (*Avena sativa* L.) under integrative use of microbial and synthetic source of nitrogen. J. Saudi Soc. Agric. Sci..

[98] Ministerio de Agricultura, Pesca y Alimentación (2020). Anuario Estadística Agraria.

[99] Gill M., Smith P., Wilkinson J.M. (2010). Mitigating climate change: The role of domestic livestock. Animal.

[100] Hegarty R.S., Alcock D., Robinson D.L., Goopy J.P., Vercoe P.E. (2010). Nutritional and flock management options to reduce Methane output and Methane per unit product from sheep enterprises. Anim. Prod. Sci..

[101] Jones A.K., Jones D.L., Cross P. (2014). The carbon footprint of UK sheep production: Current knowledge and opportunities for reduction in temperate zones. J. Agric. Sci..

[102] Shibata M., Terada F. (2010). Factors affecting Methane production and mitigation in ruminants. Anim. Sci. J..

[103] Bach A., Terré M., Vidal M. (2020). Symposium review: Decomposing efficiency of milk production and maximizing profit. J. Dairy Sci..

[104] Thompson L.R., Rowntree J.E. (2020). Invitado Review: Methane sources, quantification, and mitigation in grazing beef systems. Appl. Anim. Sci..

[105] Cruickshank G.J., Thomson B.C., Muir P.D. (2008). Modelling Management Change on Production Efficiency and Methane Output within a Sheep Flock.

[106] Castanheira É.G., Freire F. (2013). Greenhouse gas assessment of soybean production: Implications of land use change and different cultivation systems. J. Clean. Prod..

[107] Schader C., Jud K., Meier M.S., Kuhn T., Oehen B., Gattinger A. (2014). Quantification of the effectiveness of greenhouse gas mitigation measures in Swiss organic milk production using a life cycle assessment approach. J. Clean. Prod..

[108] Finnegan W., Goggins J., Chyzheuskaya A., Zhan X. (2017). Global warming potential associated with Irish milk powder production. Front. Environ. Sci. Eng..

[109] Buddle B.M., Denis M., Attwood G.T., Altermann E., Janssen P.H., Ronimus R.S., Piñares-Patiño C.S., Muetzel S., Wedlock D.N. (2011). Strategies to reduce Methane emissions from farmed ruminants grazing on pasture. Vet. J..

[110] O’Mara F.P., Beauchemin K.A., Kreuzer M., Mcallister T.A., Rowlinson P., Steele M., Nefzaoui A. (2008). Reduction of greenhouse gas emissions of ruminants through nutritional strategies. Livestock and Global Climate Change, Proceedings of the International Conference, Hammamet, Tunisia, 17–20 May 2008.

